# Immunogenicity of Cork and Loop Domains of Recombinant *Baumannii acinetobactin* Utilization Protein in Murine Model

**Published:** 2019

**Authors:** Hamid Esmaeilkhani, Iraj Rasooli, Masoomeh Hashemi, Shahram Nazarian, Fatemeh Sefid

**Affiliations:** 1. Department of Biology, Shahed University, Tehran, Iran; 2. Molecular Microbiology Research Center, Shahed University, Tehran, Iran; 3. Department of Biology, College of Basic Sciences, Imam Hussein University, Tehran, Iran; 4. Departeman of Biology, Science and Art University, Yazd, Iran

**Keywords:** *Acinetobackr baumannii*, Immune sera, Iron, Siderophore

## Abstract

**Background:**

*Acinetobacter baumannii (A. baumannii)* is a bothersome fatal pathogen, particularly in healthcare system. Persistence and successful invasion of *A. baumannii* in vertebrate host cells largely depends on iron acquisition methods. Siderophore molecules and Iron-Regulated Outer Membrane Proteins (IROMPs) are the two essential members of iron acquisition system. Siderophores are secreted by bacteria to bind peripheral ferric iron and the IROMPs are expressed at the bacterial outer membrane as the receptor of ferric-siderophore complex. BauA is the corresponding siderophore receptor of *A. baumannii*. In this study, an attempt was made to assess the immunogenicity of antigenic domains of BauA which could be effective in iron uptake restriction and protection against bacterial invasion of the host cells.

**Methods:**

The antigenic domains of *bauA* were amplified from *A. baumannii* ATCC-19606. The PCR products were ligated into pET32a and expressed in *Escherichia coli (E. coli)* BL21 (DE3). Purification of recombinant domains was done by Nickel-Nitri-lotriacetic Acid (Ni-NTA) affinity chromatography. The recombinant domains were injected into BALB/C mice separately and in combination. Sero-reactivities of the recombinant proteins and mouse challenge tests were carried out.

**Results:**

The antibodies raised in mice could successfully recognize and bind antigenic domains. Passive immunization studies accomplished by immune rabbit serum inhibited the establishment of infection in mice.

**Conclusion:**

The results adapted from the present study disclose the protective role of functional domains of BauA, especially the cork domain, suggesting a novel recombinant immunogen candidate.

## Introduction

*Acinetobacter baumannii* (*A. baumannii)* is a strictly aerobic, gram-negative, non-motile, oxidase negative coccobacillus 1. This opportunistic pathogen is widely notorious for its hospital-acquired outbreaks which constitute a growing public-health dilemma 2,3. The major clinical syndromes are pneumonia and bacteremia, together with infections in skin and soft tissue, urinary tract and surgical sites 4. *A. baumannii* can also be the major cause of meningitis, especially in patients with ventricular draining tubes 5. Several outbreaks due to *A. baumanii* have been reported 6–8.

Furthermore, MultiDrug-Resistant (MDR) *A. baumannii* has an extraordinary capacity to develop resistance against even the most potent antimicrobialcompounds, including carbapenems; hence, main infections have become incurable within recent years 9. The common carbapenem resistance mechanisms in *A. baumannii* include acquisition of carbapenemases, β-lactam-ases which are capable of hydrolyzing carbapenems, reduced affinity of penicillin binding proteins and low permeability of outer membrane proteins 1,10,11.

Iron is a fundamental nutrient required for the bacterial growth and virulence in the host cells 12. However, as a defense mechanism, most of the iron is combined with host iron storage proteins, such as transferrin, hemoglobin and hemosiderin and is therefore unattainable for bacteria 13. To be able to establish an infection, iron-starved pathogenic bacteria must synthesize, excrete and retrieve iron-scavenging low molecular weight proteins with high affinity to Fe (III), siderophores and hereby compete with and obtain iron from the host's iron binding proteins 14. In 1994, acinetobactin, the specific siderophore of *A. baumannii*, was isolated from the iron deficient cultures of *A. baumannii* ATCC19606 and its chemical structure was suggested by way of chemical degradation, FAB-MS spectrometry, and ^1^ H- and ^13^ CNMR spectroscopy methods 15,16. During a non-enzymatic isomerization in the extracellular space, pre-acinetobactin changes to acinetobactin. While the pH range <6 is optimum for the persistence of pre-acinetobactin, acinetobactin formation needs pH >7. Such an isomerization affords two siderophores for the price of one, allowing *A. baumannii* to absorb iron over a wide pH range that is probable during the infection period 17.

In order to set up infections, expression of the acinetobactin-mediated iron acquisition system is crucial for *A. baumannii* 18,19. Another group of this system in *A. baumannii* are Iron-Regulated Outer Membrane Proteins (IROMPs), which are expressed at the bacterial surface 20. The most important IROMP in *A. baumannii* is baumannii acinetobactin utilization A protein (Bau-A), which is pivotal for uptake of acinetobactin in complex with iron 12,21.

Disruption of BauA function has been shown to exert a bacteriostatic effect under iron-restricted milieu 19,22. BauA is a monomeric protein, belonging to TonB-dependent transporter protein family. The whole protein is composed of 2 domains; a cork domain at N terminal of the protein, and a trans-membrane barrel domain at the C terminal. The 22 transmembrane β-strands form barrel domain and 11 extracellular loops connect neighbor strands at the external side of the membrane. Also, 10 periplasmic turns exist in periplasmic part of the membrane 20. Cork domain comprises 168 residues from the N terminal of BauA and acts as a plug within the barrel, occluding the opening of β-barrel. Four to five anti parallel hydrophobic β-strands make up this domain 23. Ligand binding sites containing conserved residues are determined in the cork domain, suggesting a substantial role in iron entrance. Therefore, blocking of cork could have a bacteriostatic effect. Moreover, the surface-located loops which are highly exposed to the environment suggest their role in initial binding events with Fe-siderophore complex. As the extracellular part of IROMPs, loop regions are attractive targets for antigen-antibody interaction studies 24.

In the current study, the immunogenic effect of cork and loop domains were investigated separately and also in a cork-loop mixed form.

## Materials and Methods

### Bacterial strains, plasmids and media

The bacterial strains involved in this study were *A. baumannii* (ATCC19606), *Pseudomonas aeruginosa (P. aeruginosa)* (ATCC27853), *Staphylococcus aureus (S. aureus)* (ATCC25923)*, Escherichia coli (E. coli)* (ATCC25922) and *E. coli* strain BL21 (DE3). The pET32^a^ plasmid was made by Novagen (USA). Luria-Bertani (LB) broth and LB agar used to cultivate the bacterial strains were Merck (Germany) products.

### Cloning of selected domains

The forward and reverse primers for the cork (5′-GT TATGAATTCATGGATAATTCAAAAAAACTCTAGA AC-3′)/(5′-GTGCAAGCTTAAACGGTTCATCAGC-3′) and loop (5′-ATCAGGAATTCATGTTGATGGCATAT GCGG-3′)/(5′-CTTGGTCGACAGTATCCCACTCTGC TCCAA-3′) domains were designed, respectively. A downstream His-tag sequence was used on the vector to deactivate the stop codon in both genes. Amplification of coding regions corresponding to chosen antigenic domains of BauA was done using *A. baumannii* genomic DNA. The PCR process for the two genes was carried out in a mixture composed of 50 *ng/ml* of DNA, 4 *pM* of forward and reverse primers, 0.4 *mM* of dNTP mix, 3 *mM* of MgCl_2_, 1XPCR buffer and 4 *U* Taq DNA polymerase (Cinnagen Tehran, Iran) in 20 *μl* on a thermal cycler (Techne Gradient Staffordshire, UK). The procedure proceeded in the following path: initial denaturation at 94*°C* for 5 *min* and 35 cycles of 1 *min* at 94*°C*, 1 *min* at 60*°C*, 1.5 *min* at 72*°C*, and a final 5 *min* at 72*°C*.

After electrophoresis in 1% *w/v* agarose gel, PCR purification kits (Bioneer Daejeon, Korea) were applied to purify PCR products. *EcoRI* (forward) and *HindIII* (reverse) endonucleases (Fermentas Vilnius, Lithuania) undertook digestion of the cork fragment and for the loop fragment *EcoRI* (forward) and *SalI* (reverse) were responsible. The pET32^a^ vector was digested with the same enzymes of the corresponding genes. Then, the sticky ended vector and PCR products were purified using gel extraction kit. In either case, in total 40 *ng* of vector and 25 *ng* insert were mixed with 2.5 *U* of T4 DNA ligase (Fermentas Vilnius, Lithuania) and T4 ligation buffer in a 25 *μl* reaction mixture and incubated at 12*°C* for 16 *hr*. The recombinant DNA was transformed into the expression host, *E. coli* BL21 (DE3) component cell. Then, the recombinant clones were selected on LB plates containing ampicillin (50 *μg/ml*). The plasmid-insert constructs were isolated *via* plasmid extraction kits and the existence of the recombinant genes was validated by clone PCR and sequencing.

### Expression and purification of selected domains

For both domains, the process continued as follows: bacteria were grown in LB broth containing 50 *μm/ml* of ampicillin and the cultures were aerated on a shaker at 37*°C* to mid-log phase (OD_600_ of 0.6).

To maximize protein expression, 1 *mM* IPTG was added and further cultures were incubated for 4 *hr*. The cells were then harvested by centrifugation at 5000×*g* for 10 *min* at 4*°C*. The cell pellets were re-suspended in lysis buffer (100 *m M*NaH_2_PO_4_, 10 *mM* Tris.HCl, 8 *M* urea), followed by the addition of lysozyme at a 1 *mg/ml* concentration, and were sonicated 5 times for 1 *min* at 30 *s* intervals. After centrifugation at 12000 *rpm* at 4*°C* for 20 *min*, the pellet was collected for further investigation on 12% sodium dodecyl sulfate polyacrylamide gel electrophoresis (SDS-PAGE).

The proteins were purified in batch under denaturing conditions with Ni-NTA agarose (Qiagen) using buffers of different pH, all containing 8 *M* urea. Recombinant proteins were eluted using buffer E (pH=4.5). The denaturant (8 *M* urea) was removed by step wise dialysis in a Spectra/Por 2 dialysis bag (Spectrum). Finally, the proteins were dialyzed twice against phosphate-buffered saline (PBS).

The protein fractions were concentrated through freeze-drying. Densely purified proteins were estimated by the Bradford method using Bovine Serum Albumin (BSA) concentrations as standard 25.

### Western blot analysis

In order to evaluate the specificity of the recombinant domains, analysis of western blot was carried out with an Anti-His-Tag antibody. Protein samples were first electrophoresed on a 12% SDS-PAGE gel, then electro blotted into a nitro cellulose membrane at a constant current of 300 *mA* at 4*^o^C* for 1.5 *hr*. The membrane was blocked with 3% BSA with gentle shaking for 30 *min* at room temperature. The membrane was then washed 3 times with PBS-T (PBS + 0.05% Tween-20, pH=7.4) and mouse anti-His-tag mo-noclonal antibody (Abcam, USA) was added at 1:1,000 dilution for 1 *hr* at 37*°C*. Subsequently, the membranes were incubated with goat anti-mouse IgG Horseradish Peroxidase (HRP) (RAY, Biotech) at a dilution of 1:5000 for 1 *hr*. Finally, HRP staining solution (containing DAB and H_2_O_2_) led to detection of recombinant protein.

### Animal immunization

Female BALB/c mice of 4–6 weeks old (20–25 *g*) and male New Zealand White rabbits were purchased from the Razi Institute (Tehran, Iran). Animals were kept under standard conditions in the animal care facility of Shahed University. The animal care rule was ethically verified by Shahed University. For each case (cork, loop and cork-loop cocktail), mice were divided into 4 groups (5 mice per group). As new vaccination strategy comprising the simultaneous co-administration by the nasal and parenteral routes of a multicomponent vaccine formulation in BALB/C and HBsAg-transgenic mice was reported to increase the immune response 26, the same strategy and the strategies recommended by others 27 were used to immunize our test animals by both intraperitoneal and subcutaneous routes of administration. Groups were immunized on days 0, 15 and 30 *via* intraperitoneal and subcutaneous routes at the same time to reach the highest level of immunity. In order to immunize, 20 *μg* of purified protein were mixed with the same volume of adjuvant and injected to the mice. While the first dose was accompanied with complete Freund’s adjuvant, subsequent doses contained incomplete adjuvant. The other 5 mice constituted the negative control group and were injected with PBS. The experimented rabbits were immunized in a similar process with 100 *μg* of recombinant proteins and the same amount of adjuvant. Blood samples were collected 7 days after the third injection.

### Sero reactivity tests

Antibody titration was determined with indirect Enzyme-Linked Immunosorbent Assay (ELISA). Microtiter plates (Nunc) were coated with 5 *μg* of antigen (per well) together with 100 *μl* of coating buffer (bicarbonate/carbonate 100 *mM*, pH=9.6), overnight at 4*°C*. The wells were washed three times with PBS-T (PBS buffer 1X+0.05% Tween 20). Serially diluted sera (from 1:250 to 1:100000) were added to the wells and each plate was incubated for 1 *hr* at 37*°C*. Wells were rewashed with PBS-T. Primary antibodies were detected by adding 100 *μl* of HRP-conjugated anti-mouse or anti-rabbit antibodies to the wells for 30 *min* at 37*°C*. Finally, TMB, the chromogenic substrate, was added and the reaction was stopped with H_2_SO_4_.

### Whole-cell ELISA

In order to confirm the specificity, anti-cork immune serum was selected and blotted against whole-cell extracts of *A. baumannii* (ATCC19606), *P. aeruginosa*) ATCC27853), *E. coli* (ATCC25922), and *S. aureus* (ATCC25923). The bacteria were grown under iron-limiting conditions, settled down at the bottom of sterile tubes and washed two times with sterile PBS. Bacteria were then diluted using 100 *μl* of coating buffer (bicarbonate/carbonate 100 *mM*, pH=9.6) and dried onto ELISA plates at 4*°C* overnight. The wells were supplemented with 100 *μl* of blocking buffer and incubated at 37*°C* for 1 *hr* and then rinsed with PBST. Serial dilutions of immune and un-immune mice-raised sera ranging from 1/100 to 1/1000 were prepared and added to the wells (100 *μl* per well) and the process was followed by 4 *hr* incubation at 37*^o^C*. Binding of mouse sera was detected by using 100 *μl* of anti-mouse immunoglobulin G (IgG)-HRP conjugate (RAY, Biotech) in each well, then the plates were incubated at 37*°C* for 1 *hr*. After washing, 100 *μl* of citrate buffer containing 0.06% (*W/V*) of O-phenylenediamine dihydrochloride (OPD) (SIGMA) and 0.06% (*V/V*) hydrogen peroxide were added to each well and incubated at room temperature for 15 *min*. The reaction was stopped with 100 *μl* of 2MH_2_SO_4_ and the OD_492_ was read on a microplate reader (Bio-Rad).

### Determination of LD_50_ and challenge test

To calculate the 50% lethal Dose (LD_50_) in mice, *A. baumannii* dilutions in a range between l0^4^ to l0^9^ CFUs were prepared. Dilutions were intraperitoneally injected into six groups of BALB/c mice, 5 animals per group. For animal challenge test, immunized mice were dispensed into three random groups of five mice. Intraperitoneal injection of 10^ 8^, 10^9^ and 10^10^ CFU of *A. baumannii* to groups put them into challenge. LD_50_ was defined as the bacteria that are sufficient to kill 50 percent of a population of animals within 48 *hr*.

### Passive immunization

Four groups of healthy mice (5 mice per group) were selected. The first two groups were injected with 200 *μl* of immune rabbit serum at serial dilutions ranging from 1/250 to 1/10000 mixed with *A. baumannii* at 10^9^ and 10^10^CFU concentrations, following incubation at 37*°C* for 30 *min*. A 10^5^ CFU of *S. aureus* with LD_50_ of 10^4^ was used to monitor the specificity of immune response against recombinant protein domains. *S. aureus* was mixed with 200 *μl* of pure immune rabbit serum and injected into mice in group 3. Group 4 was injected with only 200 *μl* of rabbit immune serum as control.

### Investigation of A. baumannii establishment

At the same time with the death of control mice, injected mice were dissected and their liver and spleen were sterilely removed, homogenized and analyzed for the presence of *A. baumannii*. 1 *gr* of each homogenized organ was suspended in 10 *ml* of PBS. After that, 100 *μl* of each suspension was cultured on LB agar and incubated at 37*^o^C*. After 16 *hr*, bacterial colonies were counted and further tested for the identification of *A. baumannii*. Liver and spleen from mice injected only with rabbit immune serum were used as control.

### Statistical analysis

Data were reported as mean±SD and statistical analysis was performed using One-Way ANOVA (SPSS 16.0). The significance (p<0.01) of differences was assessed by post hoc comparison of means using lowest significant differences (Dunkan).

## Results

### Gene cloning, protein expression and purification

**Figure 1.**
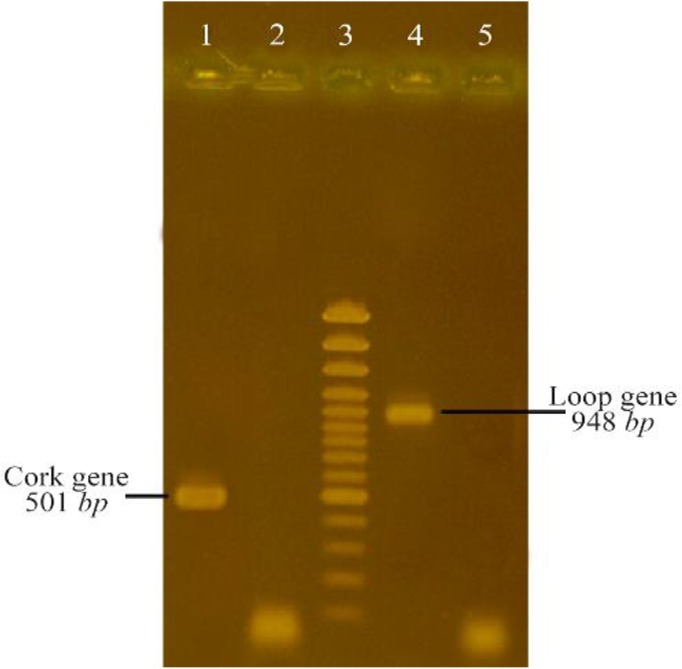
PCR amplifications of cork and loop DNA segment from BauA Lane 1: PCR product of cork, Lane 2: negative control (a PCR product without template), Lane 3:100 *bp* DNA plus ladder, Lane 4: PCR product of loop, Lane 5: negative control.

**Figure 2.**
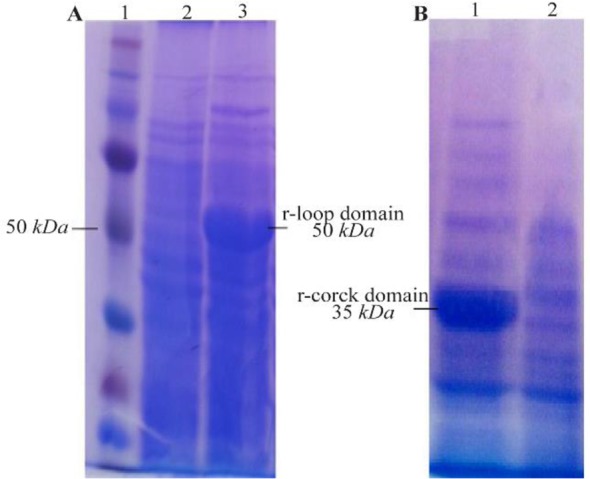
SD-PAGE analysis of the recombinant, A) loop and, B) cork domain expression. A) Lane 1: standard protein molecular weight marker, Lane 2: un-induced control, Lane 3: expression of recombinant loop domain induced with 1 *mM* IPTG, B) Lane 1: un-induced control, Lane 2: expression of recombinant cork domain.

**Figure 3.**
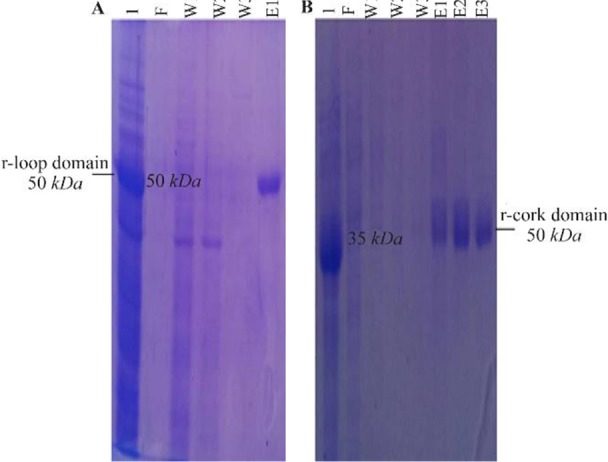
SDS-PAGE analysis of purification of recombinant (a) loop and (b) cork domain. (a) Lane 1: expression of recombinant loop domain, Lane F: unbound protein flow, Lanes W1–W3: column washed with buffer, Lane E1: column washed with elution buffer. (b) Lane 1: expression of recombinant cork domain, Lane F: unbound protein flow, Lanes W1–W3: column washed with buffer, Lanes E1–E3: column washed with elution buffer.

**Figure 4.**
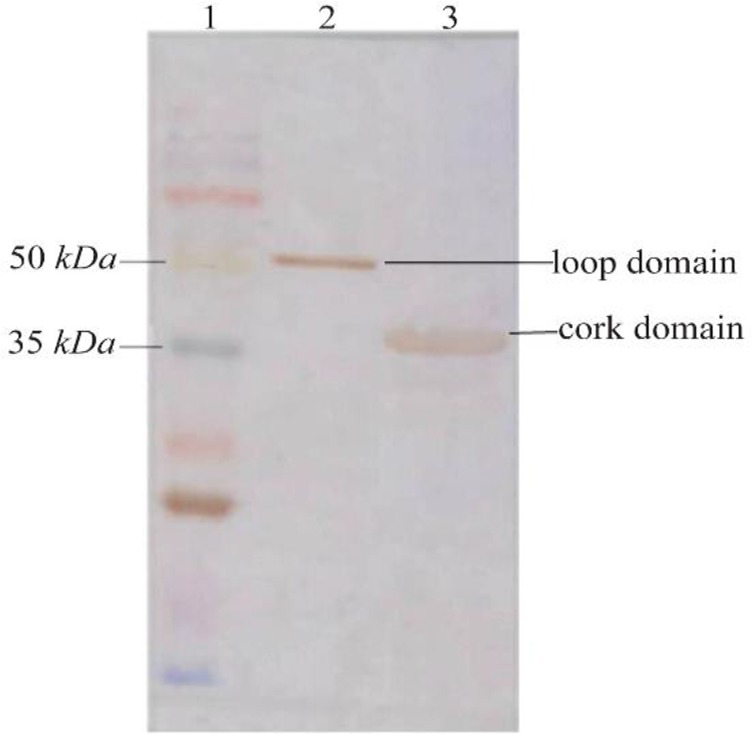
Western blot analysis of the recombinant loop and cork domains from BauA. Lane 1: molecular weight marker, Lane 2: purified loop domain, Lane 3: purified cork domain

The 948 *bp* and 501 *bp* DNA fragments encoding loop and cork domains (respectively) of BauA protein were amplified successfully (Figure 1) and cloned into pET32^a^ expression vector. Over-expression in BL21 (DE3) led to the formation of corresponding 50 *kDa* and 35 *kDa* protein bands in SDS-PAGE gel (Figure 2). After successful purification (Figure 3) and dialysis steps, the identity of the proteins was verified *via* western blotting using anti-6X His tag antibodies (Figure 4).

### Immune response

**Figure 5.**
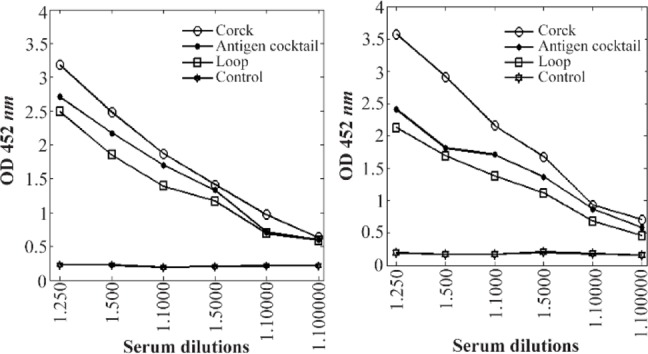
Mice (left) and rabbit (right) antibody titers against cork and loop domains of BauA.

**Figure 6.**
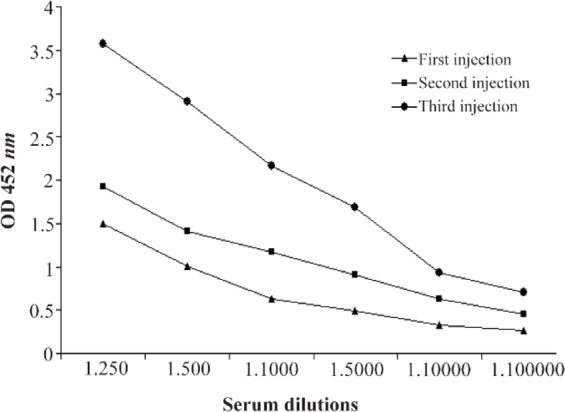
Mice antibody titers against cork domain during three injections

ELISAs demonstrated that vaccination against selected cork and loop domains of BauA protein elicited significant levels of antibodies, whereas control mice had no detectable antigen-specific antibody (p<0.001; Student’s t test). The highest immune response in BALB/c mice was achieved after the third booster injection. The highest level of IgG between different injections was detected in case of cork domain. Mice and rabbit antibody titers against selected domains are presented in figure 5. Mice antibody titers against cork domain during three injections are shown in figure 6.

### Whole-cell ELISA

**Figure 7.**
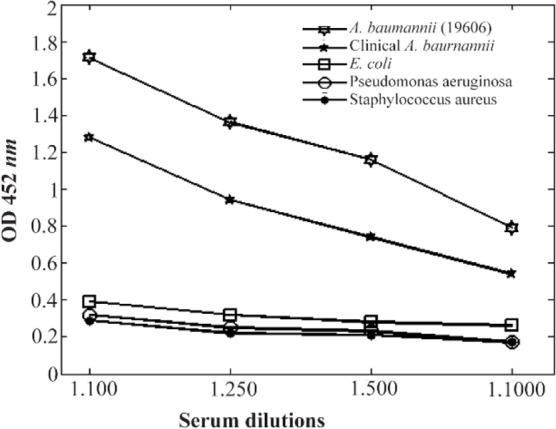
Antibody titers against whole-cell extracts of some gram −/+ strains. Mice anti-cork serum was used as antiserum against whole cells.

To investigate the specificity of the proteins, five different strains were grown under iron limitation in batch cultures and tested in whole-cell ELISAs, using anti-cork antibodies raised against *A. baumannii* 196-06. The antibodies reacted strongly with strains 19606 and also an acceptable reaction was seen with the clinical isolate. Some very weak reaction was observed with strains *E. coli*, *P. aeruginosa* and *S. aureus* (Figure 7). A similar reaction was also seen with these bacteria grown with excess iron.

### LD_50_ determination and animal challenge test

LD_50_ was determined as 10^8^ *CFU/ml* at intraperitoneal injection and mice mortality was recorded for two consecutive post-challenge days. In challenge studies, the immunized mice survived in all groups while the non-immune control groups died within 24–48 *hr*.

### Tracing bacterial infection

No bacterial colony was detected in immunized mice spleen and liver samples; however, samples from common control group revealed 16×10^4^/*g* and 23.5× 10^4^/*g* bacteria in spleen and liver cultures, respectively.

### Passive immunization

Administration of 10^9^ CFU from fresh live *A. baumannii* mixed with pure immune rabbit sera led to the survival of mice groups, while all higher sera dilutions did not impart protection.

## Discussion

It has been shown that denatured forms of outer membrane proteins of gram-negative bacteria are able to stimulate mice immune system 28,29. IROMPs, which are expressed under iron-deficient conditions, have long been considered as desirable targets for vaccine development 30.

BauA is the most important *A. baumannii* IROMP, containing conserved regions in functional domains at the cell surface 31. BauA is involved in iron uptake of *A. baumannii* to survive in harsh environments and during host infection. Hence, functional blockade of the protein could have a cidal effect on the pathogen 18.

Here, the immunogenic capability of the recombinant cork and loop domains of BauA from *A. baumannii* 19606 was investigated *in vivo*. Although the selected domains were cloned, expressed and purified under denaturing conditions 32,33, after a dialysis step, renatured forms of proteins were injected to the animals. Immunogenicity of the recombinant domains was inspected by injecting BALB/c mice and rabbits. The antibodies produced against injectants could successfully recognize and bind them. Immunized animals challenged with bacterial doses higher than *A. baumannii* LD_50_ (10^8^) survived. BauA is highly conserved in amino acid sequence and 3D structure of ligand binding sites of its functional outer membrane regions 20. Most of the functional conserved amino acids are located in plug domain and outer membrane loops. The functional sites on the surface of protein structure include cork domain and a part of barrel covering L5, L6, L7, L8 and L9. Therefore, it could be inferred that these regions are the most important functional sites of the protein. The important issue is accessibility of the antibodies to cork domain which in normal conditions is located within periplasm. The immunity generated thereby against this region could be explained by hypothesizing random or occasional exposure of this domain. This exposure facilitates their accessibility to antibodies produced against them.

In the present study, ELISAs showed the highest level of antibodies raised regarding cork domain. Our earlier bioinformatics study also introduces the cork domain at the beginning of protein sequence as the most conserved unit, with a high level of surface accessibility 23. Sequence-based and structure-based B-cell epitope prediction tools show that the cork domain holds the majority as well as the best B cell epitopes in BauA structure 23. Despite antigenic index plots which demonstrated that regions containing loops have a high antigenic propensity among BauA 23, in the current study, this domain held the lowest level of antigenic capacity *in vivo*. It could be due to the specific *in vivo* interactions between several loop structures in this domain. The cocktail of cork-loop domains showed an average level of protection among three injectants. It could be deduced that the presence of loop domains has diminished the antigenicity of cocktail during a negative interference. Moreover, acidic nature of BauA as well as its localization is an attractive factor for B-cell responses 34. B-cell epitope prediction survey has suggested that the best B cell epitopes in BauA are located in amino acids 26–191 of cork domain and amino acids 321–635 of part of the barrel domain including L4–L9. Therefore, these regions are selected as suitable vaccine candidates 23.

Passive immunization with rabbit anti-BauA antiserum also protected mice from the following infection. Post-injection bactericidal assay revealed 16×10^4^/*g* and 23.5×10^4^/*g* bacteria in spleen and liver cultures of un-immune group, respectively. The number of bacterial colonies was not significant in immune groups, showing the role of humoral immunity raised against *A. baumannii* infection. In accordance with our studies, Larrie *et al* showed that passive immunization with serum raised against an *E. coli* O157:H7 IROMP protects rabbits from subsequent infection 30.

Previously, the immunogenicity of whole BauA protein 33 was investigated and also a recombinant type was constructed by the coding regions from cork domain to loop 9 of *bauA* gene which revealed significant protection levels after the third injection dose 35.

BauA is highly prevalent in pathogenic strains of *A. baumannii* 18; hence, BauA based developed vaccines could be effective against all pathogenic strains of the bacterium. Immune serums were also used in whole-cell ELISAs using the five strains described earlier. This antiserum did not react significantly with any strains except strain 19606 and its clinical isolate, showing a specific bind of anti-BauA antiserum.

## Conclusion

In conclusion, the results adapted from the present study disclose the protective role of functional domains of BauA, especially the cork domain, suggesting a novel recombinant vaccine candidate.
